# Parliament and the Profession

**Published:** 1916-03-04

**Authors:** 


### PARLIAMENT AND THE PROFESSION.

### Maudsley County Asylum.

Replying to Mr. King, who asked whether the Lunacy Board of Control had at present any supervision over the Maudsley County Asylum at Denmark Hill, Mr. Tennant said that the Board did not exercise supervision over any military hospital. Visits that they have paid to temporarily adapted hospitals for nerve-shaken soldiers—within which category Maudsley Hospital falls—have been made at the request of the military authorities.

### The "Mauretania."

That huge floating hospital, the s.s. *Mauretania,* is being returned to her owners. She has been on hire by the Government since May 1915, and completed her last voyage on January 25, 1916. Since then she has been prepared for further service as a hospital ship, but in view of altered circumstances it has now been decided, according to a statement by Dr. Macnamara, that she can be dispensed with. It is not considered desirable to publish the cost of the ship.

### 3rd Western General Hospital.

The Army accounts are not kept in such a form as to admit of arriving at the weekly cost of a military hospital per bed, and Mr. Forster could not therefore answer Mr. Haydn Jones's question with regard to the cost per bed per week at the 3rd Western General Hospital, which is an administrative unit composed of many different buildings and contains 2,000 beds. It has a staff of some thirty-two medical officers, who draw the Army pay of their ranks, and of these nineteen are local members of the medical profession, to whom it is open to pursue private practice.

### Accommodation for Nerve-shaken Soldiers.

No further facilities are being offered through the Board of Control for the accommodation and treatment of nerve-shaken soldiers, as no further accommodation can be spared. But Mr. Tennant stated that, at the request of the War Office, the completion of the new Hampshire Asylum at Park Prewett is being expedited, with a view to its being equipped and staffed by the military authorities as a temporary war hospital. In the view of the Government the existing arrangements for the care of uncertifiable cases of soldiers suffering from nerve disturbance are satisfactory.

### National Health Insurance Affairs.

With reference to the Committee of Inquiry, which was dealt with in The Hospital of February 26 (p. 485), several questions have been asked regarding the personnel of the committee. There are, Mr. Montagu explained, three women members, and it is not intended to vary the composition of the committee by the addition either of another representative of approved societies or an Irish labour representative. Replying to a question dealing with another branch of Insurance Act administration, Mr. Roberts stated that in the interests of economy the activities of district insurance commitees had been suspended in many areas.

### R.A.M.C. Officers Unfit for Foreign Service.

Temporarily commissioned officers of the Royal Army Medical Corps under forty-five years of age, and unfit for foreign service, are not permitted to retain their commissions after the period of service for which they engaged has expired. No further commissions are being given to men under forty-five unless they are physically fit for active service. Mr. Tennant, in making the above statement, added that it was hoped by these methods to set free men who are physically capable of undertaking general military duties and who have hitherto been employed in war hospitals in this country or have been engaged in private practice.

### Other Matters of Interest.

Tuberculous Soldiers.—Replying to Mr. Astor, Mr. Tennant stated that the total number of cases of soldiers invalided for tuberculosis which were dealt with by the Chelsea Boards in 1915 was 2,770.

Blind Soldiers.—According to a statement made by the Under-Secretary for War, fifty-nine privates, four corporals, one sergeant, and one company sergeant-major have been rendered totally blind in the present war. All these will receive pensions.

Midwives (Scotland) Act.—The Board under this Act has now been set up. Replying to Mr. Watt, Mr. McKinnon Wood states that there appeared to be no likelihood of any immediate or substantial claim upon the local authorities for any deficit between the Board's income and expenditure.

Sanitary Section of the R.A.M.C.—Mr. Charles Duncan, being under the impression that men attested under the Derby scheme possessing special qualifications and experience as sanitary inspectors were being passed over, asked whether an Army Order had been issued instructing commanding officers of the Royal Army Medical Corps to give preference to unattested men in making appointments to advertised positions in the Sanitary Section. Mr. Tennant said that no such Order had been issued.

